# Forecasting model of *Corylus*, *Alnus*, and *Betula* pollen concentration levels using spatiotemporal correlation properties of pollen count

**DOI:** 10.1007/s10453-015-9418-y

**Published:** 2015-12-14

**Authors:** Jakub Nowosad, Alfred Stach, Idalia Kasprzyk, Elżbieta Weryszko-Chmielewska, Krystyna Piotrowska-Weryszko, Małgorzata Puc, Łukasz Grewling, Anna Pędziszewska, Agnieszka Uruska, Dorota Myszkowska, Kazimiera Chłopek, Barbara Majkowska-Wojciechowska

**Affiliations:** 1 Institute of Geoecology and Geoinformation, Adam Mickiewicz University, Dzięgielowa 27, 61-680 Poznań, Poland; 2 Department of Environmental Biology, University of Rzeszów, Zelwerowicza 4, 35-601 Rzeszów, Poland; 3 Department of Botany, University of Life Sciences in Lublin, Akademicka 15, 20-950 Lublin, Poland; 4 Department of General Ecology, University of Life Sciences in Lublin, Leszczyńskiego 58, 20-950 Lublin, Poland; 5 Department of Botany and Nature Conservation, University of Szczecin, Felczaka 3c, 71-412 Szczecin, Poland; 6 Laboratory of Aeropalynology, Faculty of Biology, Adam Mickiewicz University, Umultowska 89, 61-614 Poznań, Poland; 7 Department of Plant Ecology, University of Gdańsk, Wita Stwosza 59, 80-308 Gdańsk, Poland; 8 Department of Clinical and Environmental Allergology, Jagiellonian University Medical College, Śniadeckich 10, 31-531 Kraków, Poland; 9 Faculty of Earth Sciences, University of Silesia, Będzińska 60, 41-200 Sosnowiec, Poland; 10 Department of Immunology, Rheumatology and Allergy, Faculty of Medicine, Medical University, Pomorska 251, 92-215 Łódź, Poland

**Keywords:** Aerobiology, Allergenic pollen, Betulaceae, Forecast, Random forest, Spatiotemporal models

## Abstract

The aim of the study was to create and evaluate models for predicting high levels of daily pollen concentration of *Corylus*, *Alnus*, and *Betula* using a spatiotemporal correlation of pollen count. For each taxon, a high pollen count level was established according to the first allergy symptoms during exposure. The dataset was divided into a training set and a test set, using a stratified random split. For each taxon and city, the model was built using a random forest method. *Corylus* models performed poorly. However, the study revealed the possibility of predicting with substantial accuracy the occurrence of days with high pollen concentrations of *Alnus* and *Betula* using past pollen count data from monitoring sites. These results can be used for building (1) simpler models, which require data only from aerobiological monitoring sites, and (2) combined meteorological and aerobiological models for predicting high levels of pollen concentration.

## Introduction

*Corylus* L. (hazel), *Alnus* Mill. (alder), and *Betula* L. (birch) belong to the order Fagales Engl. and the family Betulaceae Gray (Bremer et al. 2009). These trees are very common in the Northern Hemisphere (Kornas and Medwecka-Kornas 2002). The dominant species from this family in Poland are *Alnus glutinosa*, *Alnus incana*, and *Betula pendula*; less common are *Betula pubescens*, *Corylus avellana*, and their cultivars. The start and length of the *Corylus* and *Alnus* pollen seasons are very variable from year to year. Their pollen season in Poland usually begins some time between early February and late March and lasts on average for 30 days (*Corylus*) and 26 days (*Alnus*). The *Betula* pollen season occurs between the middle of April and the middle of May and lasts for approximately 18 days. Its pollen season start and duration are less variable than those of *Corylus* and *Alnus* (Nowosad et al. 2015). Furthermore, the daily and annual pollen counts of *Corylus*, *Alnus*, and *Betula* vary greatly (Nowosad et al. 2015).

*Corylus*, *Alnus*, and *Betula* pollen are well known for their allergenic properties (Viander and Koivikko 1978), and the occurrence of allergic reactions is connected with pollen concentration levels. According to Rapiejko et al. (2007), the first allergy symptoms are seen during exposure to a concentration of $35 \hbox { pollen}/\hbox {m}^{3}$ of air for *Corylus*, $45 \hbox { pollen}/\hbox {m}^3$ of air for *Alnus* , and $20 \hbox { pollen}/\hbox {m}^3$ of air for *Betula*. Allergy symptoms in all subjects were noted at concentrations of 80, 85, and $75 \hbox { pollen}/\hbox {m}^3$ of air, respectively, for *Corylus*, *Alnus*, and *Betula*. Sensitization rates to tree species of the family Betulaceae in Poland are high: *Corylus*, 22.3 %; *Alnus*, 22.8 %; and *Betula*, 27.7 % (Heinzerling et al. 2009). The pollen allergens from *Corylus*, *Alnus*, and *Betula* are structurally and immunochemically similar. Thus, cross-reactions are very likely between allergens of the *Betula* family (Valenta et al. 1991).

One of aerobiology’s objectives is to develop models enabling the prediction of pollen concentration in the air (Rodriguez-Rajo et al. 2006). Forecast models of pollen concentrations have many practical applications. They are highly important for allergy sufferers because predictions can allow them to undertake appropriate treatment. Models could also be useful in agriculture, forestry, and many fields of science. Most of the published results are based on the relationship between pollen season characteristics or on pollen count and meteorological conditions (Bringfelt et al. 1982; Cotos-Yáñez et al. 2004; Castellano-Méndez et al. 2005; Rodriguez-Rajo et al. 2006; Hilaire et al. 2012). Other models have been built based on an operational weather forecast system (Vogel et al. 2008) and the System for Integrated modeLling of Atmospheric coMposition (SILAM) (Sofiev et al. 2013). In Poland, Latałowa et al. (2002) delimited the major meteorological parameters as a basis for future forecast modeling of the atmospheric *Betula* pollen concentration in Gdańsk; Puc (2012) built an artificial neural network model of the relationship between *Betula* pollen and meteorological factors in Szczecin; and Myszkowska (2013) predicted *Betula* pollen season characteristics in Kraków.

Aerobiological surveys have been focused either on the statistical relationship between pollen count and meteorological variables or on a deterministic representation of pollen dispersion. A recent study showed that pollen counts are highly inert, temporally and spatially (Nowosad et al. 2015). Models based on this property would be relatively simple, because they do not require data other than that of pollen concentration. The forecasts would require data only from aerobiological monitoring sites and could be almost completely automated. The aim of this study was to create and evaluate *Corylus*, *Alnus*, and *Betula* pollen concentration level predictions based on previous pollen count values from given sites. To the best of our knowledge, there are no reports in the literature regarding how these kinds of models perform in practice.

## Materials and methods

### Study area

**Fig. 1:**
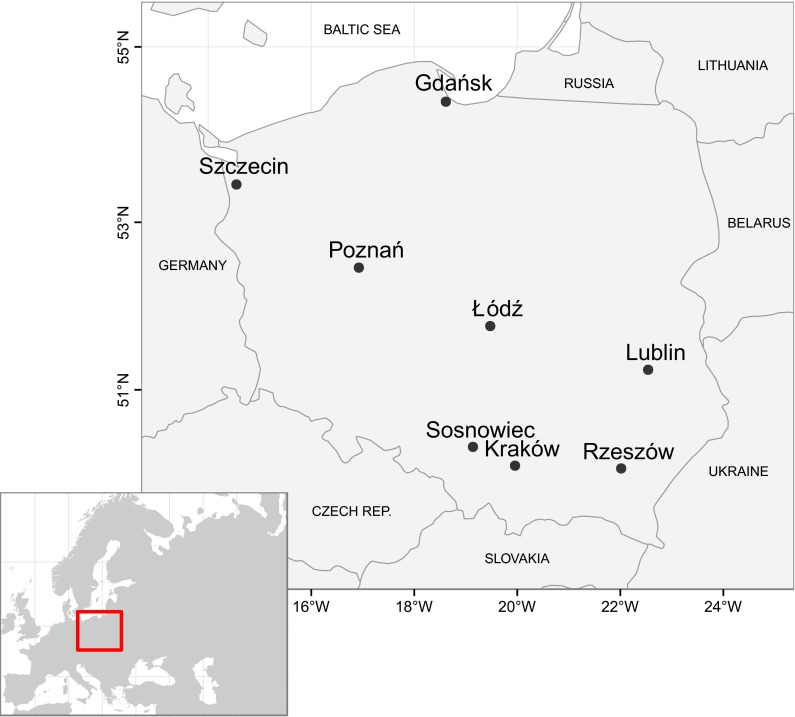
The location of the sites used for the study of pollen concentration levels prediction

The studies were conducted in eight cities in Poland (Gdańsk, Kraków, Lublin, Łódź, Poznań, Rzeszów, Sosnowiec, and Szczecin) and covered six years of measurement (2003–2005, 2009–2011) (Fig. 1; Table 1). The measurements from years 2006–2008 were not available for all of the sites. Therefore, incomplete data were not included.

Poland is a country in Central Europe, extending 649 km from north to south and 689 km from east to west. It is a lowland country with an average elevation of 173 m and a surface area of 312,679 km$^{2}$, only 3.1 % of which is higher than an elevation of 500 m. There are five topographic zones in Poland. The order of the zones from north to south is as follows: the Baltic coastal plains, the lake region, the central lowlands, the uplands, and the mountains (Sudeten and Carpathian ranges). Agricultural land covers approximately 60 % of the surface area of the country, and forest, bush, and wooded land cover about 30 %. Built-up and urbanized areas occupy approximately 5 % of the total area (Dmochowska 2013). All of the studied cities are agglomerations surrounded mainly by forests and farmlands. However, the proportion of land use classes around each city is different. Gdańsk lies on the coast of the central Baltic Sea, with sea to the east and agricultural lands or forests to the west and south; Szczecin is located between Dąbie Lake to the northeast, forests to the southeast, and agricultural areas to the southwest; Poznań and Łódź are surrounded by forests and agricultural areas; Lublin is surrounded by agricultural areas; Rzeszów lies between forests to the north and agricultural areas to the south, east, and west; Kraków and Sosnowiec are distinguished by a larger proportion of urban areas.

Poland has a temperate continental climate. The effects of Atlantic masses of air and the proximity of the Baltic Sea are felt in Gdańsk and Szczecin. Poznań, Łódź, Sosnowiec, and Kraków are located in a transition zone between oceanic and continental air masses. The climate of Rzeszów and Lublin is influenced by continental air masses. In addition, Kraków, Sosnowiec, and Rzeszów lie near the Carpathian Mountains, which affect their climate (Blazejczyk 2006). A two-sample Kolmogorov-Smirnov test yielded no significant differences ($D = 0.08$, $p\hbox { value} = 0.17$) between the daily temperatures for the six years of the study (2003–2005, 2009–2011) and a 30-year time series of measurements (1983–2012).

**Table 1:** Characteristics of the study sites: latitude, longitude, and altitude of the aerobiological monitoring sites; population of the cities; and mean temperatures recorded at local meteorological stations

City	$\lambda$ (DD)	$\phi$ (DD)	Altitude (a.s.l.)	Population (in thousands)	Mean temperature ($^{\circ }\hbox {C}$) (1983–2012)				
					Annual	January	February	March	April
Gdańsk	18.6131	54.3856	10	460	7.42	−1.40	−1.40	1.78	6.66
Kraków	19.9559	50.0637	212	758	8.47	−1.90	−1.11	3.12	8.94
Lublin	22.5402	51.2437	198	348	7.81	−2.66	−2.22	1.93	8.31
Łódź	19.4748	51.7715	216	719	8.43	−1.62	−1.05	2.80	8.76
Poznań	16.9243	52.4671	91	551	8.96	−0.62	−0.18	3.59	9.08
Rzeszów	22.0160	50.0293	209	182	8.46	−2.10	−1.28	2.87	8.79
Sosnowiec	19.1389	50.2972	252	214	8.47	−1.50	−0.81	3.08	8.72
Szczecin	14.5478	53.4395	30	409	8.96	0.18	0.57	3.74	8.60

### Aerobiological data

Daily average pollen counts were measured by a volumetric spore trap of the Hirst design (Hirst 1952), according to the recommendations of the European Aerobiology Society’s Working Group on Quality Control Galán et al. (2014). Traps were located 12 m above ground level (Rzeszów) or higher. Two different pollen counting methods were used. Pollen grains were counted along 12 vertical transects using the methods outlined by Stach (2000) (Gdańsk and Rzeszów), or along four horizontal transects using the method recommended by the Spanish Aerobiology Network (Kraków, Lublin, Łódź, Poznań, Sosnowiec, Szczecin) (Galán et al. 2007). Cariñanos and Emberlin (2000) reported that both methods follow similar trends and provide close approximations to the pollen count from the entire slide. The pollen concentration was expressed as the number of $\hbox {grains}/\hbox {m}^3$ of air per 24 h (Comtois 1998).

### Dataset creation

All the calculations were carried out using R software packages (R Core Team 2014; Kuhn 2014; Liaw and Wiener 2002). The pollen season limits of *Corylus*, *Alnus*, and *Betula* at each location and for each year were calculated using the 90 % method (Nilsson and Persson 1981). In this method, a season starts when 5 % of the total catch has been achieved and ends when 95 % has been reached. For each taxon, the earliest day of pollen season start and the latest day of pollen season end based on all of the data were used as the temporal scope.

**Fig. 2:**
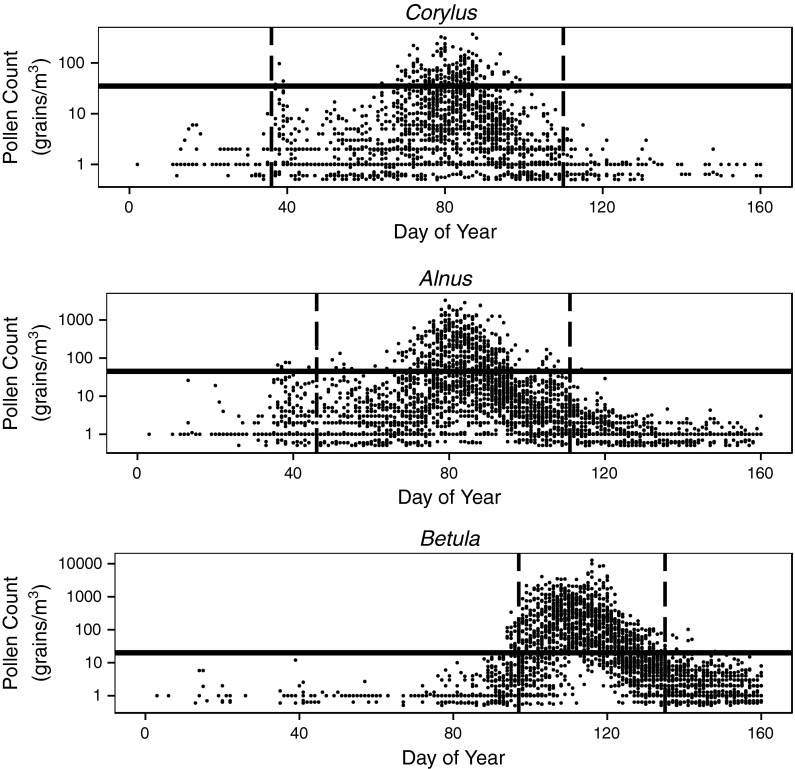
Pollen count of *Corylus*, *Alnus*, *Betula* by day of year for all of the analyzed sites in years 2003–2005 and 2009–2011 on a logarithmic scale. *Vertical lines* indicate the temporal scope of analysis for each taxon. *Horizontal lines* separate the two pollen concentration levels of low and high: (35 g/m^3^ for *Corylus*, 45 g/m^3^ for *Alnus*, 20 g/m^3^ for *Betula*)

The aim of this work was to forecast the pollen count level of allergenic risk. For each taxon, two levels of concentration were distinguished: low and high. The ranges of concentration level values were different for each taxon. The concentration levels were as follows: *Corylus*, low 0–35 $\hbox {grains}/\hbox {m}^{3}$ and high >35 grains/m^3^; *Alnus*, low 0–45  grains/m^3^ and high >45 grains/m^3^; and *Betula*, low 0–20 grains/m^3^ and high >20 grains/m^3^ (Table 2). Threshold values were based on first symptom values for patients allergic to these taxa (Rapiejko et al. 2007).

### Statistical modeling

By using a stratified random split to divide the datasets, the distribution of the outcome in the training and test sets was preserved. Two subsets were created:

- Training set, used for training a model and choosing its optimal parameters (2/3 of cases)
- Test set, used only to evaluate the model on data not present during previous stages (1/3 of cases)

Most of the machine learning algorithms expect equal instances of each class. Thus, the imbalance between classes can have a significant impact on the quality of the model. The dataset was slightly imbalanced in the case of *Betula*, and highly imbalanced for *Corylus* and *Alnus* (Table 2). Up-sampling was used to reduce this class imbalance. This technique imputes additional data to improve balance across the classes. Training sets were sampled with replacements to create equal class distribution. All of the original training data were left intact, and additional samples were added to the minority classes with replacements. However, the test sets were not changed, since they should reflect the class imbalance. This is important to obtain reliable estimates of a model’s performance (Kuhn and Johnson 2013).

**Table 2:** Absolute and relative number of days with given pollen concentration levels for the individual taxa and location

Taxon	City	Low	High
*Corylus*	Gdańsk	432 (98.63 %)	6 (1.37 %)
*Corylus*	Kraków	404 (92.24 %)	34 (7.76 %)
*Corylus*	Lublin	395 (90.18 %)	43 (9.82 %)
*Corylus*	Łódź	421 (96.12 %)	17 (3.88 %)
*Corylus*	Poznań	420 (95.89 %)	18 (4.11 %)
*Corylus*	Rzeszów	413 (94.29 %)	25 (5.71 %)
*Corylus*	Sosnowiec	406 (92.69 %)	32 (7.31 %)
*Corylus*	Szczecin	406 (92.69 %)	32 (7.31 %)
*Alnus*	Gdańsk	344 (89.58 %)	40 (10.42 %)
*Alnus*	Kraków	334 (86.98 %)	50 (13.02 %)
*Alnus*	Lublin	310 (80.73 %)	74 (19.27 %)
*Alnus*	Łódź	332 (86.46 %)	52 (13.54 %)
*Alnus*	Poznań	308 (80.21 %)	76 (19.79 %)
*Alnus*	Rzeszów	332 (86.46 %)	52 (13.54 %)
*Alnus*	Sosnowiec	333 (86.72 %)	51 (13.28 %)
*Alnus*	Szczecin	292 (76.04 %)	92 (23.96 %)
*Betula*	Gdańsk	111 (50 %)	111 (50 %)
*Betula*	Kraków	80 (36.04 %)	142 (63.96 %)
*Betula*	Lublin	88 (39.64 %)	134 (60.36 %)
*Betula*	Łódź	96 (43.24 %)	126 (56.76 %)
*Betula*	Poznań	71 (31.98 %)	151 (68.02 %)
*Betula*	Rzeszów	105 (47.3 %)	117 (52.7 %)
*Betula*	Sosnowiec	100 (45.05 %)	122 (54.95 %)
*Betula*	Szczecin	79 (35.59 %)	143 (64.41 %)

Previous research in Poland showed that there is usually an increase—delayed on average by 1–3 days—in the correlation of pollen count between the pairs of monitoring sites (Nowosad et al. 2015). This is due mainly to prevailing winds from the west toward the east in this latitude zone of the Northern Hemisphere (Rossby waves) and the movement of atmospheric fronts. Levels of *Corylus*, *Alnus*, and *Betula* pollen concentration were used as the outcome data. The independent variables were the previous 4 days’ pollen counts from all of the sites:

1 $$\begin{aligned} \hbox {PollenConcentrationLevel}_{\mathrm{siteA}_{t}}\sim & {} \hbox {PollenCount}_{\mathrm{siteA}_{t-1}} + \hbox {PollenCount}_{\mathrm{siteA}_{t-2}} \\ &\quad +\hbox {PollenCount}_{\mathrm{siteA}_{t-3}} + \hbox {PollenCount}_{\mathrm{siteA}_{t-4}} \nonumber \\&\quad +\hbox {PollenCount}_{\mathrm{siteB}_{t-1}} + \cdots + \hbox {PollenCount}_{\mathrm{siteH}_{t-4}} \end{aligned}$$

Random forest (Breiman 2001) was used to predict the pollen concentration levels of *Corylus*, *Alnus*, and *Betula*. Preliminary studies showed that a random forest model’s performance is comparable to other techniques, such as support vector machines and boosting trees. Furthermore, valuable information about random forest results could be obtained using, for example, variable importance. A random forest model has one tuning parameter: the number of randomly selected predictors to choose from at each split ($m_{\mathrm{try}}$). A total of 100 iterations of the bootstrap were applied as the re-sampling scheme to select the optimal values of the model’s tuning parameter. A series of models were fit to the training sets. For each model, the optimal parameter value was obtained based on specificity: the rate that days with high concentration were predicted correctly:

2 $$\begin{aligned} \text {Specificity} = \frac{\hbox {CP}_{\mathrm{high}}}{\hbox {All}_{\mathrm{high}}} \end{aligned}$$

where $\hbox {CP}_{\mathrm{high}}$ is the number of correctly predicted days with high concentration and $\hbox {All}_{\mathrm{high}}$ is the total number of days of high concentration.

The general effect of predictors on each model was calculated. Variable importance was estimated by looking at the increase in prediction error when data for a given variable were changed, while all the other variables remained constant (Breiman 2002; Liaw and Wiener 2002). Afterward, the variable importance was scaled to values between 0 and 100.

### Evaluation of the models’ performance

The final 24 models (3 taxa $\times$ 8 cities) were applied to generate predictions based on the test sets. Model predictions were then compared with the observed data in the test sets. A confusion matrix, unweighted Kappa statistic, sensitivity, specificity, and balanced accuracy were used to describe the performance of the models. The Kappa statistic is:

3 $$\begin{aligned} \text {Kappa} = \frac{O - E}{1 - E} \end{aligned}$$

where *O* is the observed accuracy and *E* is the accuracy expected to be achieved based on the marginal totals of the confusion matrix. The Kappa statistic ranges from $-$1 to 1. A value of 0 indicates no agreement between the observed and predicted classes, while a value of 1 indicates perfect agreement. Negative values rarely occur and indicate that “the prediction is in the opposite direction of the truth” (Kuhn and Johnson 2013). The sensitivity is defined as the rate that days with low concentration are predicted correctly:

4 $$\begin{aligned} \text {Sensitivity} = \frac{\hbox {CP}_{\mathrm{low}}}{\hbox {All}_{\mathrm{low}}} \end{aligned}$$

where $\hbox {CP}_{\mathrm{low}}$ is the number of correctly predicted days with low concentration and $\hbox {All}_{\mathrm{low}}$ is the total number of days with low concentration.

Balanced accuracy helps to reduce the impact of imbalanced classes on a model’s evaluation. It is defined as follows:

5 $$\begin{aligned} \text {Balanced}\,\text {accuracy} = \frac{\text {Sensitivity} + \text {Specificity}}{\text {2}} \end{aligned}$$

Additionally, Mann–Whitney *U* test (Mann and Whitney 1947) was used to compare the weather conditions (precipitation and temperature) between true and false predictions of low and high pollen concentration levels of *Corylus*, *Alnus*, and *Betula*.

## Results

**Table 3:** A summary of *Corylus*, *Alnus*, and *Betula* models results for training set at each location

Taxon	City	Kappa	Accuracy	Sensitivity	Specificity
*Corylus*	Gdańsk	0.99	1.00	0.99	1.00
*Corylus*	Kraków	0.97	0.99	0.97	1.00
*Corylus*	Lublin	0.96	0.98	0.96	1.00
*Corylus*	Łódź	0.99	1.00	0.99	1.00
*Corylus*	Poznań	0.98	0.99	0.98	1.00
*Corylus*	Rzeszów	0.98	0.99	0.98	1.00
*Corylus*	Sosnowiec	0.97	0.98	0.97	1.00
*Corylus*	Szczecin	0.98	0.99	0.98	1.00
*Alnus*	Gdańsk	0.94	0.97	0.94	1.00
*Alnus*	Kraków	0.97	0.98	0.97	1.00
*Alnus*	Lublin	0.92	0.96	0.93	0.99
*Alnus*	Łódź	0.94	0.97	0.95	1.00
*Alnus*	Poznań	0.91	0.96	0.93	0.99
*Alnus*	Rzeszów	0.94	0.97	0.95	1.00
*Alnus*	Sosnowiec	0.96	0.98	0.96	1.00
*Alnus*	Szczecin	0.90	0.95	0.93	0.97
*Betula*	Gdańsk	0.61	0.81	0.81	0.81
*Betula*	Kraków	0.84	0.92	0.94	0.91
*Betula*	Lublin	0.82	0.91	0.92	0.90
*Betula*	Łódź	0.81	0.90	0.91	0.90
*Betula*	Poznań	0.81	0.91	0.94	0.88
*Betula*	Rzeszów	0.77	0.89	0.88	0.90
*Betula*	Sosnowiec	0.71	0.86	0.85	0.87
*Betula*	Szczecin	0.74	0.87	0.91	0.84

**Table 4:** A summary of *Corylus*, *Alnus*, and *Betula* models results for test set at each location

Taxon	City	Kappa	Balanced accuracy	Sensitivity	Specificity
*Corylus*	Gdańsk	0.00	0.50	1.00	0.00
*Corylus*	Kraków	0.54	0.76	0.97	0.55
*Corylus*	Lublin	0.68	0.84	0.97	0.71
*Corylus*	Łódź	0.23	0.59	0.99	0.20
*Corylus*	Poznań	0.00	0.50	1.00	0.00
*Corylus*	Rzeszów	0.49	0.79	0.96	0.62
*Corylus*	Sosnowiec	0.49	0.74	0.97	0.50
*Corylus*	Szczecin	0.41	0.65	0.99	0.30
*Alnus*	Gdańsk	0.60	0.76	0.98	0.54
*Alnus*	Kraków	0.74	0.84	0.98	0.71
*Alnus*	Lublin	0.71	0.89	0.90	0.88
*Alnus*	Łódź	0.86	0.91	0.99	0.82
*Alnus*	Poznań	0.72	0.86	0.95	0.76
*Alnus*	Rzeszów	0.66	0.83	0.96	0.71
*Alnus*	Sosnowiec	0.67	0.81	0.97	0.65
*Alnus*	Szczecin	0.65	0.84	0.90	0.77
*Betula*	Gdańsk	0.76	0.88	0.92	0.84
*Betula*	Kraków	0.81	0.92	0.96	0.88
*Betula*	Lublin	0.72	0.86	0.86	0.87
*Betula*	Łódź	0.67	0.84	0.88	0.81
*Betula*	Poznań	0.75	0.88	0.83	0.92
*Betula*	Rzeszów	0.67	0.83	0.77	0.90
*Betula*	Sosnowiec	0.81	0.90	0.85	0.95
*Betula*	Szczecin	0.62	0.81	0.77	0.85

### Model

The start of the earliest *Corylus* pollen season was on day 36 of the year, and its latest season end was on day 110 of the year. For *Alnus*, these start and end dates ranged from days 46 to 111 of the year, and for *Betula* they ranged from days 97 to 135 of the year. Data only from these periods were used for model creation and evaluation (Fig. 2).

**Fig. 3:**
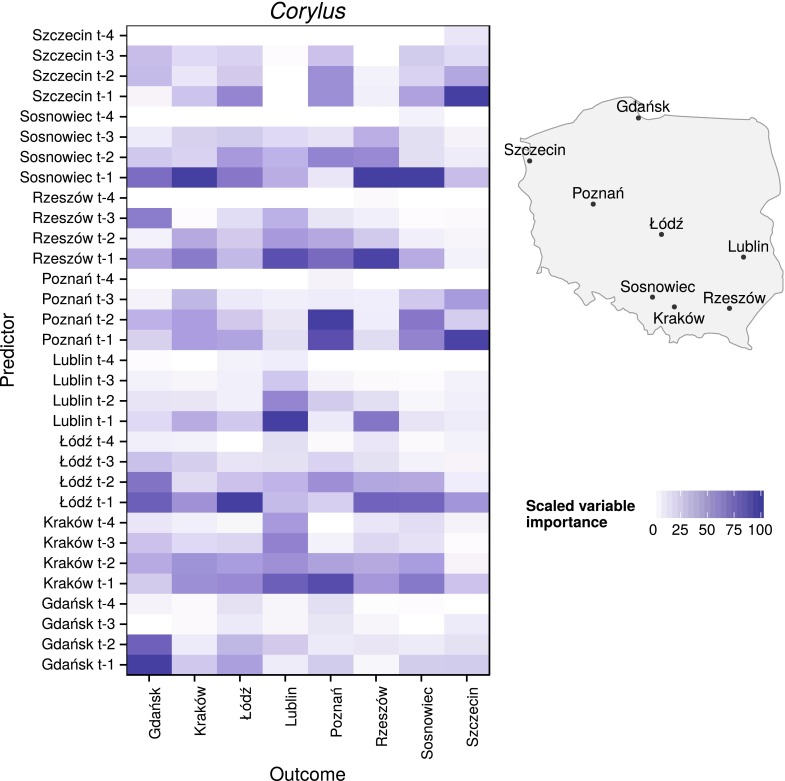
Scaled variable importance of each predictor (pollen count data with 1-day lag ($t-1$), 2-day lag ($t-2$), 3-day lag ($t-3$), 4-day lag ($t-4$) at given sites) for *Corylus* in each location. For better spatial relations recognition, a schematic map with measurement sites was provided

**Fig. 4:**
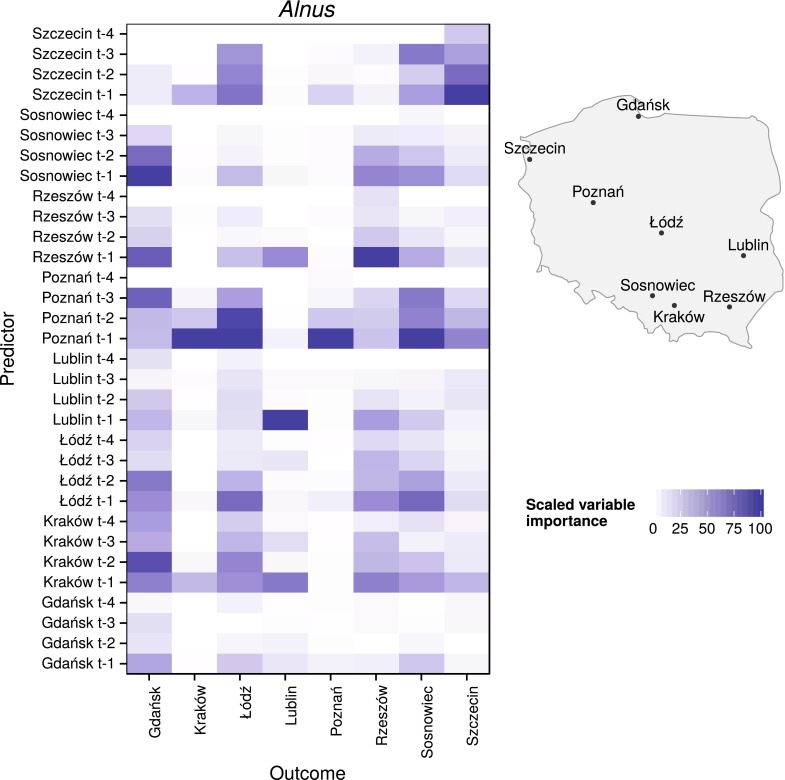
Scaled variable importance of each predictor (pollen count data with 1-day lag ($t-1$), 2-day lag ($t-2$), 3-day lag ($t-3$), 4-day lag ($t-4$) at given sites) for *Alnus* in each location. For better spatial relations recognition, a schematic map with measurement sites was provided

**Fig. 5:**
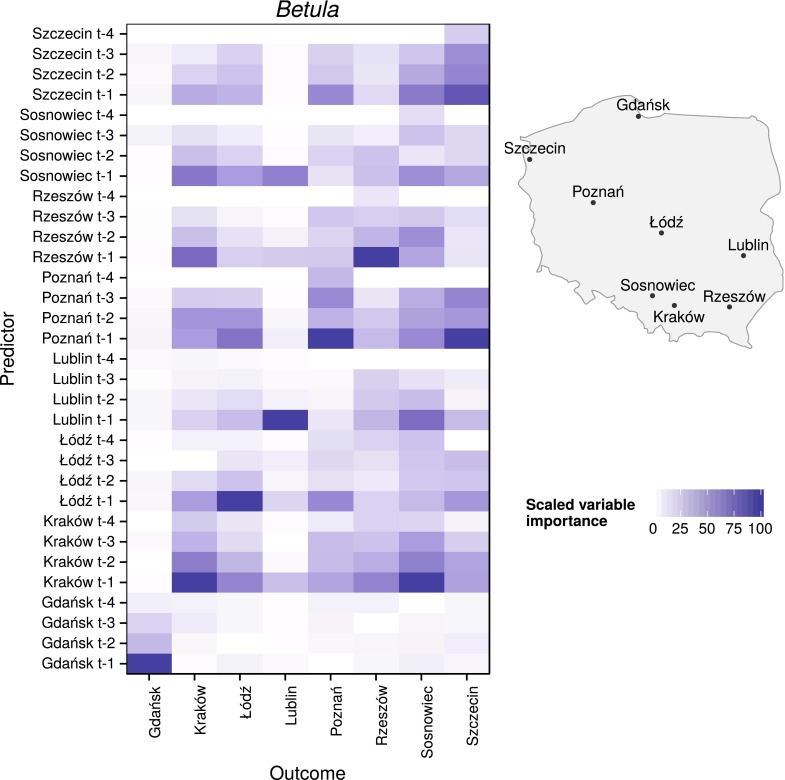
Scaled variable importance of each predictor (pollen count data with 1-day lag ($t-1$), 2-day lag ($t-2$), 3-day lag ($t-3$), 4-day lag ($t-4$) at given sites) for *Betula* in each location. For better spatial relations recognition, a schematic map with measurement sites was provided

**Fig. 6:**
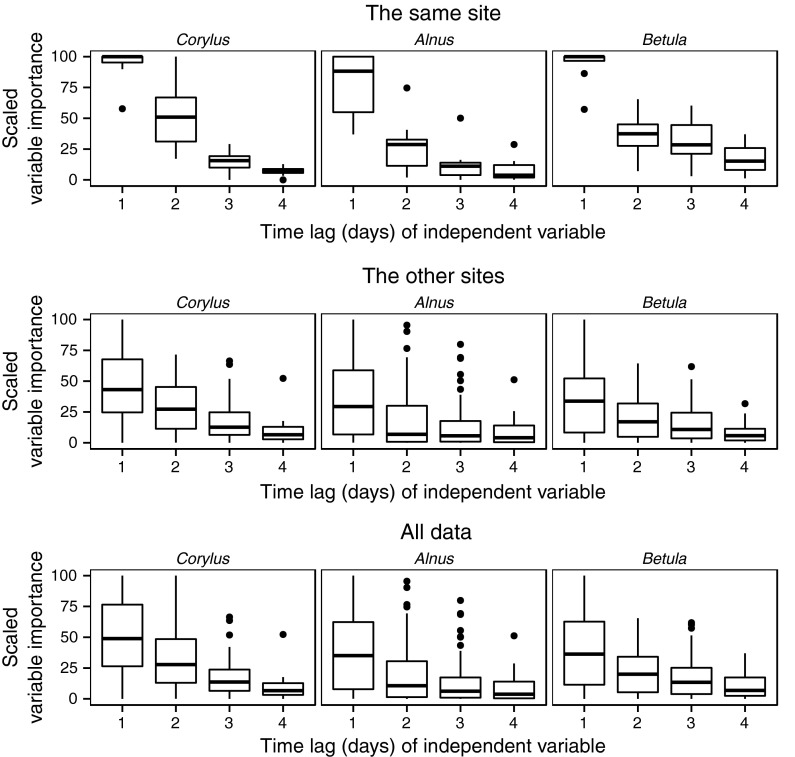
Variations of the variables importance for each lag for *Corylus*, *Alnus*, and *Betula* models. The same site: only data from the same site. The other sites: all data except from the same site, and all data

**Fig. 7:**
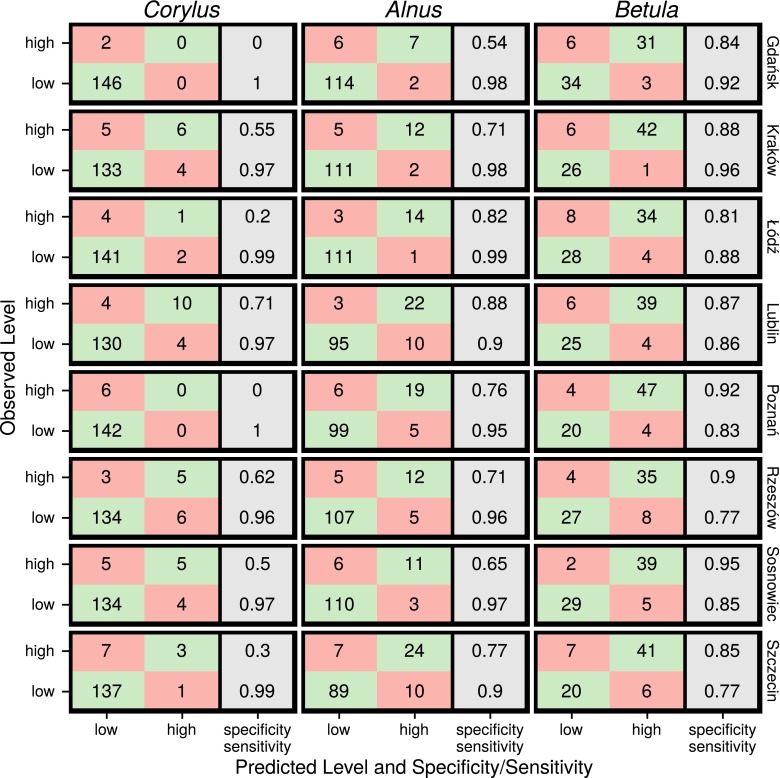
Confusion matrices and specificity/sensitivity for test sets of pollen concentration level prediction for *Corylus*, *Alnus*, and *Betula* at each location

Twenty-four final models were created. Table 3 shows a summary of model results for training sets. Most (13 of 16) *Corylus* and *Alnus* models had a specificity value equal to 1. Only in the cases of Lublin, Poznań, and Szczecin was the *Alnus* model’s specificity slightly lower: 0.99, 0.99, and 0.97, respectively. Kappa statistics were also very high for the *Alnus* and *Corylus* models, with values ranging from 0.90 (for *Alnus* in Szczecin) to 0.99 (for *Corylus* in Gdańsk and Łódź). Model performance values based on the *Betula* training datasets were lower. However, in the majority of the models, the specificity values were still very high: between 0.81 for Gdańsk and 0.91 for Kraków. Kappa values varied noticeably, from 0.61 for Gdańsk to 0.84 for Kraków.

### Variable importance

Variable importance for *Corylus*, *Alnus*, and *Betula* models shared similar temporal and spatial properties. The values of pollen counts from one day before were the most important variable, while the values from 4 days before were the least. Moreover, variables from the same site were the most important in the majority of the models.

For six of eight of the *Corylus* models, the most important variable was the pollen count from one day (Gdańsk, Lublin, Sosnowiec, and Łódź) or 2 days (Poznań) before. Only in the case of models of pollen concentration levels in Kraków and Rzeszów was the most important independent variable from a different city: Sosnowiec in both cases. In *Corylus* models, the low importance of pollen concentration inputs from 4 days before was noticeable (Figs. 3, 6).

Variable importance for *Alnus* models was the least uniform. Only in half of the models (Lublin, Poznań, Rzeszów, and Szczecin) was the most important variable from the same city as the output. Also, in four of eight models (Poznań, Łódż, Kraków, Sosnowiec), the most important variable was the pollen count in Poznań from one day before. Exceptionally, the most important variable for predicting pollen concentration levels in Gdańsk was data from Sosnowiec. The low impact of variables from 4 days before was also apparent (Figs. 4, 6).

Variable importance for *Betula* models showed some regularities. In six of eight models, the most important variables were pollen concentration from a day before at the same site as the outcome. In addition, the pollen count values at the same site from 2 to 3 days before had a visible influence. The order of variable importance was different in two models. The most important variable for the *Betula* model in Sosnowiec was the pollen concentration value in Kraków from one day before; and the pollen concentration value in Poznań from one day before was the most important variable for the Szczecin model. Moreover, models for Gdańsk and Lublin were built based mainly on the values from the same site. In the rest of the models, many independent variables were important. Inputs from Gdańsk also had a small impact on the rest of the models. In addition, for most of the models, variables from 4 days before had either little importance or no importance at all (Figs. 5, 6).

### Performance of the models

A confusion matrix, Kappa statistic, sensitivity, specificity, and balanced accuracy were used to evaluate the models on the test sets (Fig. 7; Table 4). *Corylus* models showed the lowest Kappa values. In two cases (Gdańsk and Poznań), Kappa statistics were equal to 0. Only the model of Lublin had a substantial Kappa value (0.68).; it correctly predicted 10 of 14 cases with a high pollen concentration (Fig. 7; Table 4).

The Kappa statistics also proved important for *Alnus* models, with an average value of 0.7. The minimum Kappa value was for the Gdańsk model (0.6), and the maximum was for the Łódź model (0.86). However, most of the *Alnus* models had a specificity lower than the *Betula* models. Only models for Lublin and Łodź specificity exceeded 0.8 (0.88, 0.82, respectively). The lowest specificity was found for the model for Gdańsk: 0.54. For the test set, the model of pollen concentration group of Gdańsk predicted correctly only 7 of 13 cases of high pollen concentration (Fig. 7; Table 4).

Based on th given criteria, models of *Betula* were the most reliable. Their average Kappa was 0.73, with a minimum value of 0.62 for Szczecin and a maximum of 0.81 for Kraków and Sosnowiec. Moreover, all of the specificity values for *Betula* models exceeded 0.8. In all of the *Betula* models, specificity values were higher than the Kappa statistic (Fig. 7; Table 4).

True and false predictions were compared with temperature and precipitation. The results indicated that the rainfall was connected with a false prediction of high level in Alnus ($p\hbox { value}=0.0018$) and Betula ($p\hbox { value}=0.0000002$) models. However, this relation was not found in Corylus ($p\hbox { value}=0.12$) models. Additionally, true and false predictions were compared to the day-to-day changes in precipitation and temperature. The results showed that the final models were robust to changes in the precipitation; however, predictions of high level of Corylus ($p\hbox { value}=0.0016$), Alnus ($p\hbox { value}=0.01$), and Betula ($p\hbox { value}=0.001$) are sensitive to the changes in temperature.

## Discussion

One of the main goals in aerobiological models is to predict pollen concentration levels which can trigger the onset of allergic symptoms. Stepwise multiple regression (Bringfelt et al. 1982; Myszkowska 2013), additive logistic models, partially linear models (Cotos-Yáñez et al. 2004), artificial neural networks (Castellano-Méndez et al. 2005), ARIMA models (Rodriguez-Rajo et al. 2006), and stochastic gradient boosting (Hilaire et al. 2012) have been used in aerobiological studies aimed at pollen concentration prediction. Most pollen predictive modeling studies have focused on the impact of meteorological variables (such as temperature, humidity, precipitation, wind direction, and speed), on pollen season start and duration, and on pollen concentration (Bringfelt et al. 1982; Cotos-Yáñez et al. 2004; Castellano-Méndez et al. 2005; Rodriguez-Rajo et al. 2006; Hilaire et al. 2012; Myszkowska 2013). Only Castellano-Méndez et al. (2005) attempted to forecast the level of allergenic risk associated with *Betula* using previous *Betula* pollen and meteorological information. However, to the best of our knowledge, empirical predictive models have not used pollen count values from other sites before now. Previous research shows that *Corylus*, *Alnus*, and *Betula* pollen concentration are correlated not only in time, but also in space (Nowosad et al. 2015).

Nowadays, daily pollen concentration data are a result of manual pollen counting. Thus, information about the pollen count from the previous day is not available fast enough to be used for predicting levels of pollen concentration. However, there are many efforts to create a semiautomatic and automatic systems for counting airborne pollen (Boucher et al. 2002; Holt and Bennett 2014). As a result of these studies, it should be possible to obtain information about the pollen data from the day before quickly enough to be used by a forecast system.

In this study, random forest (Breiman 2001) was used to predict the pollen concentration level of *Corylus*, *Alnus*, and *Betula* using a spatiotemporal correlation of pollen count values at the given sites. The use of random forest presents a distinct advantage with respect to classical statistical methods: the ability to process complex, nonlinear relationships between predictors (Recknagel 2001). The algorithm of random forest is based on the ensemble of a large number of decision trees (Breiman 2001). Consequently, random forest has the advantage of tree-based models over artificial neural networks or support vector machines: interpretability (Geurts et al. 2009). A random forest model can be explained by visualization of decision trees or by using measures of variable importance.

The models of *Alnus* and *Betula* had, at the least, considerable values of model evaluation statistics. More than 81 % of events with high *Betula* pollen concentration could be predicted at each of the given sites. The models for *Corylus* showed low values of performance statistics in most cases. There are a few possible explanations for this. Firstly, there were insufficient events with high pollen concentration levels in the training/test sets and small values of *Corylus* pollen count generally. In the years 2003–2005 and 2009–2011, high Corylus pollen counts occurred only between 6 and 43 times (26 on average), and *Corylus* pollen concentration was lower than 19 grains/m$^3$ on 90 % of the analyzed days. Secondly, *Corylus* models could be highly overfit, possibly due to a relatively short time series. The start and course of *Corylus* pollen season strongly depends on the type of habitat. *Corylus* pollen season starts sooner in sunny locations and cities, where only a few degrees above zero are enough to start the pollination. On the contrary, *Corylus* pollen season starts later in the sunless sites or forests. Therefore, its pollen season lasts longer than *Alnus* or *Betula* (Puc and Kasprzyk 2013). The pollen season of *Alnus* starts on average one weeks after *Corylus* in Poland (Nowosad et al. 2015). *Alnus* is also less common in the cities, and its start of flowering is less changeable between trees (Bugała 2000; Puc and Kasprzyk 2013). As a result, the relationship between *Corylus* pollen data in different cities is more complex than *Alnus* or *Betula*. Longer time series could reveal more information about Corylus spatiotemporal properties. Therefore, the variable importance of *Corylus* models should be interpreted with caution.

In this study, the model for *Betula* correctly forecasted between 81 and 95 % of the days with high pollen concentrations in the test set. This model performance is similar to the previous work of Castellano-Méndez et al. (2005), who used artificial neural networks for predicting whether Betula pollen concentrations exceed certain thresholds—20, 30, 70, and 80 g/m$^3$—using previous pollen and meteorological information. The artificial neural networks model predicted between 83 and 100 % of over-level pollen days on the validation set (years 2000 and 2001). Thus, the models based on previous pollen counts from several sites could serve as an alternative to models based on pollen and meteorological data from a single analyzed site.

The importance of independent variables showed a clear temporal and spatial dependency. In 23 of 24 models, variables from a day before had the largest impact. Moreover, input from the same site as the output was the most important in 16 of 24 models. In *Alnus* models, a high impact of variables from Poznań on Łódź, Sosnowiec, and Kraków was observed. This relationship was also found for *Betula* models. At the same time, in *Betula* models, aside from those at Szczecin and Sosnowiec, the input values from the nearest sites had clear importance; for example, Rzeszów and Sosnowiec for Kraków; Poznań for Łódź; Szczecin and Łódź for Poznań; and Poznań for Szczecin. One possible explanation is that neighboring stations have similar weather conditions and therefore similar pollen emission. The other explanation is influence of long-distance transport caused by the dominant westerly direction of winds in Poland. After a one-day lag, the variable importance noticeably decreases. It was found that variables from 4 days before show the least importance, with average values of 10.52, 8.58, and 8.67 % for *Corylus*, *Alnus*, and *Betula*, respectively, and the highest values did not exceed 50 %. This could be associated with the inflow of a new air mass with different physical characteristics from the previous one. In Poland, the variability of most strings of days with a single air mass type was determined to be between 1 and 3 days (Kotas et al. 2013). It should be noted that variable importance is not a measure of causation.

Pollen production and dispersion is affected by many factors: regional flora, land use, vegetation structure, topoclimate, and weather conditions. Pollen concentration in air cannot be described as a linear effect of the impact of these factors. Despite the fact that *Alnus* and *Betula* models had substantial prediction quality, some of the events of low or high pollen level were wrongly classified. This is connected mainly with unusual events, such as no pollen or low pollen concentration at monitoring sites on the days before and high pollen count at a given site. Or it may be the opposite: low pollen concentration at a given site and high pollen count at monitoring sites in the preceding days. The occurrence of these situations can be partially explained by the influence of atmospheric conditions. The pollen concentration level could be low during the rainfall and high on the next day with a dry weather. The rapid day-to-day temperature changes also can be the cause of the models errors. Additionally, the occurrence of wrongly classified cases could probably be explained by other factors, such as random local events or changes in scale smaller than those analyzed.

Gdańsk distinguished itself from the other sites. Days with high pollen concentration of *Corylus*, *Alnus*, and *Betula* were less frequent there. In most of the models, independent variables from Gdańsk had little or no importance. Moreover, the prediction quality for Gdańsk was the lowest in the *Corylus* and *Alnus* models. As has been reported previously (Nowosad et al. 2015), Gdańsk has different pollen characteristics from other Polish sites. Its northern, coastal location has an impact on the local climate, and the start of the growing season is usually delayed there.

## Conclusions

In this study, data from eight Polish monitoring sites over six years were used. The final 24 models are not necessarily the best ones in terms of prediction quality. The *Corylus* models performed poorly, which could be a mixed result of (1) insufficient events with high pollen concentration level in the training/test set, (2) highly overfit models, and (3) fast change of pollen autocorrelation drop.

On the other hand, the study has clearly shown that it is possible to predict the occurrence of days with high pollen concentration of *Alnus* and *Betula* using past pollen count data from monitoring sites. For these taxa, random forest models offer capabilities for forecasting pollen concentration levels, with substantial accuracy. The models are an alternative to pollen concentration models based on weather conditions, and they show promise as a useful source of information on high pollen concentration levels for allergists and their patients. It would thus be worthwhile to combine two groups of independent variables—meteorological and aerobiological—from several sites to improve models for predicting pollen concentrations which exceed threshold values. An analysis of longer time periods or a denser monitoring network could also result in better model quality, especially in the case of *Corylus*.
